# Meeting report on the first Sino-Dutch symposium on oncology

**DOI:** 10.1186/s40880-016-0093-3

**Published:** 2016-03-16

**Authors:** Maikel P. Peppelenbosch

**Affiliations:** Erasmus Medical Center Cancer Institute, Erasmus University of Rotterdam, PO Box 2040, 3000 CA Rotterdam, The Netherlands

**Keywords:** Meeting report, Cancer care

## Abstract

On October 31, 2015, the first Sino-Dutch symposium on oncology was organized in Guangzhou (China). The symposium revealed similarities between Chinese and Dutch efforts to improve the care of tumor patients and to create enhanced insight into the nature of cancers. In particular, it became evident for some types of cancer that immunotherapy should focus on counteracting interleukin-17-associated immunity and targeting cancer stroma. Targeting specific cancer microenvironment and stroma also opens new therapeutic options, including the use of radio-active theranostics and live tumor imaging-guided surgeries.

## Background

**Fig. 1:**
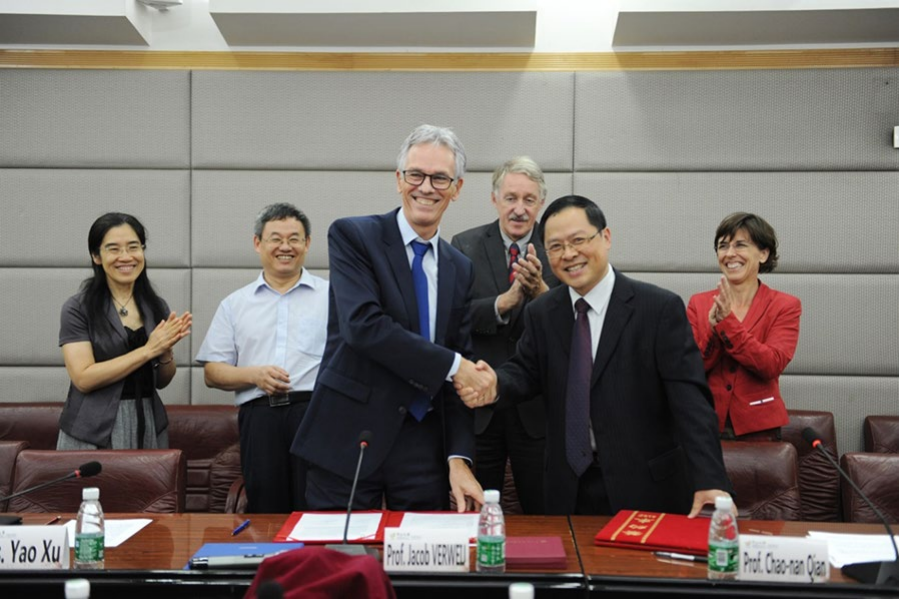
Prof. Dr. Jaap Verweij (Dean of the Erasmus Medical Center executive board; *left in the front*) and Prof. Chao-Nan (Miles) Qian (Vice President of the Sun Yat-sen University Cancer Center; *right in the front*) congratulate each other with the signing of the Sino-Dutch collaboration agreement between these two institutions. * From left to right* in the back: Ms. Yao Xu (Deputy Director, Office of International Cooperation and Exchange, Sun Yat-sen University), Prof. Limin Zheng, (Biotherapy Center, Sun Yat-sen University Cancer Center), Mr. Peter Boostma (Health, Welfare and Sport Counselor at Embassy of the Kingdom of the Netherlands in China), Mrs. Marjo Crompvoets (Consul General of the Kingdom of the Netherlands in Guangzhou)

**Fig. 2:**
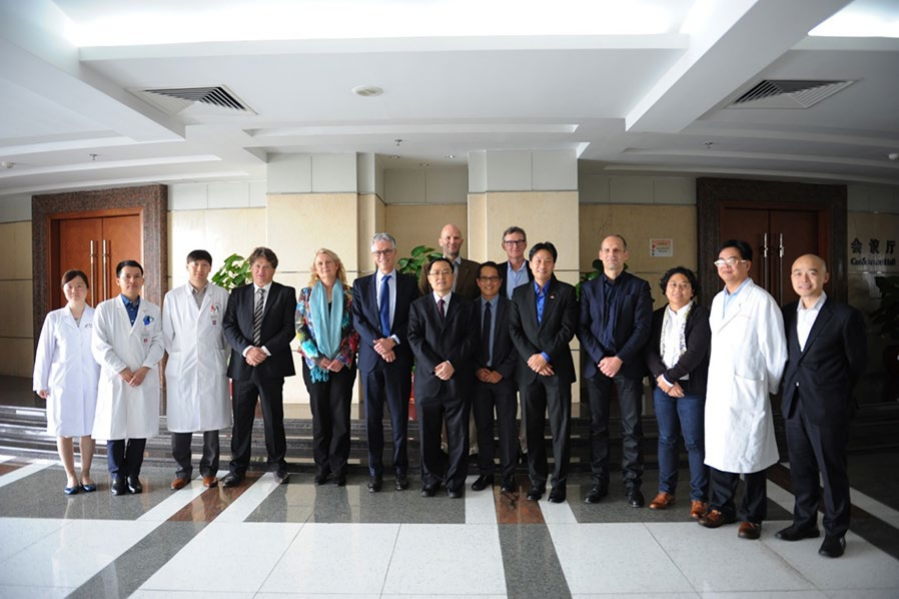
Some of the speakers at the first Sino-Dutch Symposium on Oncology. *From left to right:* Dr. Shuang Liao, Dr. Yu-Jia Zhu, Prof. Zhen-Ffeng Zhang, Prof. Jean-Phille Pignol, Prof. Marion de Jong, Prof. Jaap Verweij, Prof. Chao-Nan (Miles) Qian (*in the front*), Prof. Timo ten Hagen (*in the back*), Prof. Jose Hardillo, Prof. Clemens Löwik, Mr. Raoul Tan, Prof. Reno Debets, Mrs. Gonny Pasaribu, Prof. Li-Wu Fu, and Mr. Jingmin Kan (Assistant Counsellor for Science and Technology at Embassy of the Kingdom of the Netherlands in China)

Many societies in the present era are characterized by the unprecedented phenomenon of population aging, which imposes substantial new challenges to heath care. Perhaps the foremost of these challenges is an increased burden of tumors; and, indeed, in many societies, cancer has become the main cause of death. Hence, healthcare systems are under substantial pressure to develop novel modalities aimed at improving the diagnosis and treatment of tumors, both with respect to clinical outcome and patient quality of life. This issue is particularly true for both China and Europe, which are both subjected to substantial population aging, especially when compared with, for instance, the United States of America or India. It follows that Chinese and European health institutions should seek collaboration to develop novel avenues for combating tumors, as both face relatively similar challenges. Accordingly, collaboration was initiated between the Sun Yat-sen University Cancer Center (SYSUCC, one of the top cancer centers in China) and the Erasmus Medical Center (Erasmus MC, the largest university hospital in the Netherlands) on all aspects of clinical cancer care and research. This relationship was recently formalized through the signing of a mutual memorandum of understanding between the Erasmus MC and SYSUCC (Fig. 1). The collaboration was initiated by Prof. Chao-Nan (Miles) Qian (Vice President of SYSUCC) and Prof. Dr. Jaap Verweij (Dean of the Erasmus MC Executive Board). Florence Sand (SYSUCC) and Raoul Tan (Erasmus MC) were instrumental in arranging the administrative and logistic details involved. This collaboration promises to provide great oncological research synergy at both institutions and should prove important for the field in general. The unusual potential of the collaboration became evident at the first Sino-Dutch symposium on oncology that was held on October 31, 2015 in Guangzhou, in which eminent scientists from both institutions presented their work (Fig. 2), which in conjunction produced important new insights. This meeting report aims to convey the progress reached during the symposium to a larger audience.

## Thoracic cancer

Thoracic cancers (e.g., esophageal cancer, lung cancer, breast cancer) represent the most common cancer types not only in China but also in the patient population of the Erasmus MC. Early detection and intervention can substantially contribute to clinical outcome of such cancers. For example, proton pump inhibition therapy can substantially reduce the risk for malignant progression of esophageal cancer [1]. In my presentation (Maikel Peppelenbosch; Erasmus MC), I highlighted the importance of biochemical characterization of both premalignant and malignant tissues for guiding prevention as well as treatment of such cancers, and this plan was echoed well by data presented by Ling Cai (SYSUCC). He demonstrated that detecting mutations of the epidermal growth factor receptor (EGFR) in non-small cell lung cancer does not provide significant guidance for predicting the efficacy of EGFR tyrosine kinase inhibitors (TKIs), especially when combined with whole body radiation therapy. Remarkably, in the latter presentation, data were shown that the clinical efficacy of TKIs was independent of the actual mutation (either in the receptor tail or loss of the extracellular domain), and this observation corresponded well with my own earlier finding that kinase activity in thoracic (pre-)cancerous lesions was relatively independent of the underlying mutation status [2]. Similarly, Dr. Lan-Jun Zhang (SYSUCC) presented data that attempted to identify genetic variations in pulmonary lymphoepithelioma, but the findings were not conclusive. Taken together, these findings suggest that the current emphasis on next-generation sequencing of thoracic cancer samples for guiding personalized medicine may prove to be disappointing. This conclusion highlights how new insights can emerge from bilateral conferences like the one reported here.

## The role of the tumor microenvironment

Cases on the role of the tumor microenvironment were presented by Dr. Limin Zheng (SYSUCC) and Dr. Reno Debets (Erasmus MC), who both focused on the role of the immune system in combating tumors. Dr. Zheng presented his data on the microenvironment of hepatocellular carcinoma, showing that this environment is characterized by suppressive macrophages, which appear Th17-polarized, and thus are poor interferon γ secretors. This microenvironment, although highly pro-inflammatory, is not permissive for cytotoxic T cell responses, enabling a growing tumor to evade immunosurveillance. Alone, these data are highly interesting, as they mechanistically explain the well-established capacity of hepatocellular carcinoma to escape attack by the immune system, even in the context of copious immunoreactivity towards tumor antigens in patients with this type of cancer [3]. However, in context of the work presented by Dr. Reno Debets, these data acquire additional compelling relevance. Dr. Debets has a very efficient platform for engineering enhanced T cell receptors, which can efficiently attack a broad range of tumor types. It has often been suggested that such approaches would benefit from co-administration with ipililumimab, a biological medicine that counteracts regulatory activity. A realization emerged in the audience from Dr. Zheng’s prior presentation that, at least for hepatocellular carcinoma, anti-interleukin-17 antibodies (currently used for treating multiple sclerosis but not tumors) may be a more promising treatment strategy.

## Novel approaches to cancer eradication

Interestingly, the specific microenvironment created by tumors to escape anti-cancer immunity may also prove to be an Achilles heel for these cancers. Such specific microenvironment may result in the strong expression of peptide receptors with an otherwise much more restricted expression. As Dr. Marjon de Vos (Erasmus MC) showed in her exciting presentation, such peptide receptors are excellent targets for therapeutic peptide radionucleotides, which upon binding to their cognate ligands can kill cancer cells by emitting ionizing radiation [4]. Intriguing proof-of-principle examples were provided by Dr. de Vos, who showed that down-regulation of the receptors involved was probably incompatible with the necessity to maintain a specific microenvironment to escape immunosurveillance. As shown by Dr. Zheng, it is possible to significantly improve clinical outcomes. Indeed, the necessity of a specific tumor microenvironment was also highlighted by various other presentations. For example, Dr. Clemens Löwik (Erasmus MC) presented examples of highly advanced imaging technology capable of visualizing mesenchymal stem cells and showed important constituents of a variety of cancers. Such novel imaging modalities are likely to contribute to better care, as they allow for detection of cancer cells in the operation theater, allowing surgeons to ensure that the entire cancer is resected. This notion was further supported by data from Dr. José Angelito Ugalde Hardillo (Erasmus MC), who showed that tumor samples have specific Raman spectra. In the future, this technique may provide further specificity and provide real-time guidance to the surgeons during operation procedures.

## Drug delivery

Therapeutic efficacy, however, either through novel drugs or by conventional drugs, critically depends on the efficiency of drug delivery. New insights emerged through the synergy generated in this Sino-Dutch symposium. Dr. Timo ten Hagen (Erasmus MC) presented data obtained with intravital microscopy that conventional liposomal delivery of doxorubicin to tumor cells lacks efficacy because of slow release of the drug from the liposome, a problem potentially further compounded by liposomal processing by the tumor cells. The discovery of Dr. Xin Mei (SYSUCC), also employing live imaging, that glioblastoma cells display vascular mimicry and can apparently create potentially functional blood vessels was also presented at this symposium. Cancer cells in general benefit from the presence of blood vessels, and for clear cell renal cell carcinoma, anti-angiogenic therapy is even the treatment of choice [4]; however, efficient blood supply also provides a route for drug delivery. This notion critically depends on the functionality of these blood vessels [5]. The use of tissue factors as indicator molecules to confirm or exclude the functionality of the vessel-like structures involved was extensively elaborated on during the ensuing discussion, further illustrating the value of this type of symposium.

## Conclusions

The first Sino-Dutch symposium was unusually productive with respect to generating new insight into how cancer in aging societies can be approached. This initial success augurs very well for closer collaboration between the Erasmus MC and SYSUCC. As both centers now develop clinical implementation of proton therapy (presentation of Dr. Jean-Philipe Pignol) for tumors, further benefits and synergies will likely continue to emerge from this collaboration.
